# Dental approach for Apert syndrome in children: a systematic review

**DOI:** 10.4317/medoral.21628

**Published:** 2017-10-21

**Authors:** Andrea-Stacy López-Estudillo, Miguel-Ángel Rosales-Bérber, Socorro Ruiz-Rodríguez, Amaury Pozos-Guillén, Ángel Noyola-Frías, Arturo Garrocho-Rangel

**Affiliations:** 1 DDS, Especialidad en Estomatología Pediátrica, Facultad de Estomatología, Universidad Autónoma de San Luis Potosí, San Luis Potosí, S.L.P., México; 2 DDS, MSc, Especialidad en Estomatología Pediátrica, Facultad de Estomatología, Universidad Autónoma de San Luis Potosí, San Luis Potosí, S.L.P., México; 3 DDS, MSc, PhD, Laboratorio de Ciencias Básicas, Facultad de Estomatología, Universidad Autónoma de San Luis Potosí, San Luis Potosí, S.L.P., México; 4 DDS, Servicio de Cirugía Oral y Maxilofacial, Hospital Central “Dr. Ignacio Morones Prieto”, San Luis Potosí, S.L.P., México; 5 DDS, MSc, PhD, Especialidad en Estomatología Pediátrica, Facultad de Estomatología, Universidad Autónoma de San Luis Potosí, San Luis Potosí, S.L.P., México

## Abstract

**Background:**

Apert Syndrome (AS), or type I acrocephalosyndactyly, is a rare, congenital craniosynostosis condition resulting from missense mutations in the gene encoding fibroblast growth factor receptor 2. It is characterized by three specific clinical features: brachycephalic skull; midface hypoplasia, and limb abnormalities (syndactyly of hands and feet). The disorder exhibits variable presentations in bones, brain, skin, internal organs, and in the oral/maxillofacial region. The aim of the present paper was to show the main results from a systematic review of AS.

**Material and Methods:**

A search of the literature was performed from April to June 2016 in five electronic databases. Clinical interventional or observational studies, reviews, and case reports were included. The present systematic review was carried out strictly following PRISMA and Cochrane Collaboration criteria.

**Results:**

A total of 129 potential references were identified. After reviewing titles and abstracts, 77 of these did not meet the desired criteria and were discarded. The full text of the remaining 52 manuscripts was critically screened. Finally, 35 relevant papers were identified for inclusion in the present systematic review and classified according to topic type.

**Conclusions:**

According to the information gathered, dentistry practitioners must be able to supply an early diagnosis through the recognition of AS clinical features and provide correct oral management. Additionally, they should be integrated in a multidisciplinary medical care team in order to improve the quality of life of the affected patients.

** Key words:**Apert syndrome, acrocephalosyndactyly, craniosynostosis, skeletal dysplasias, systematic review.

## Introduction

Apert syndrome (AS), also known as acrocephalosyndactyly, is one of the rarest and most severe cranio-synostosis syndromes, accounting for about 4.5% of all craniosynostosis cases (1,2). AS was first clinically described by Baumgartner in 1842 and by Wheaton in 1894; later, it was reviewed extensively by Eugene Apert, a French Pediatrician, who published a series of nine cases in 1906 (3-5).

AS is a rare congenital autosomal dominant disorder that is characterized by severe craniosynostosis (premature closure/fusion of multiple calvarial sutures, specifically the coronal suture) and associated with cranio-facial anomalies, including symmetric 2nd to 4th digit syndactyly in hands and feet (partial or complete fusion of the skin and bones of fingers/toes, with a common nail (6); in severe cases, there may occur synostosis of the radius/humerus and shoulder and elbow joints, with ocular (shallow orbits, exophthalmia, strabismus, hypertelorism, and down-slanting palpebral fissures), ear (chronic otitis media, hearing loss), respiratory (obstructive sleep apnea, mouth breathing), skin (acne, excessive sweating), brain (ventriculomegaly, hydrocephalus), and malformations of the *corpus callosum* and/or limbic structures; in addition, some children may exhibit a mild mental/intellectual deficit, with an average Intelligence Quotient (IQ) of 74, pharyngeal (short in height), and internal organ abnormalities (gastrointestinal, cardiovascular, genitourinary) (7-10). Craniosynostosis leads to a restriction of facial-skeleton anteroposterior growth, from the glabella to the posterior fontanelle, giving rise to the characteristic cone-shaped head of AS (*acrobrachycephaly or turribrachycephaly*) (9,11). Craniofacial findings include midface/maxillary hypoplasia with Class III malocclusion, premature fusion of the fifth and sixth cervical vertebrae, flat forehead and occiput, a depressed broad nose with bulbous tip, and deviated septum (12). Buco-dental features are described in  Table 1 (4,7,9,11,13-16).

**Table 1:**
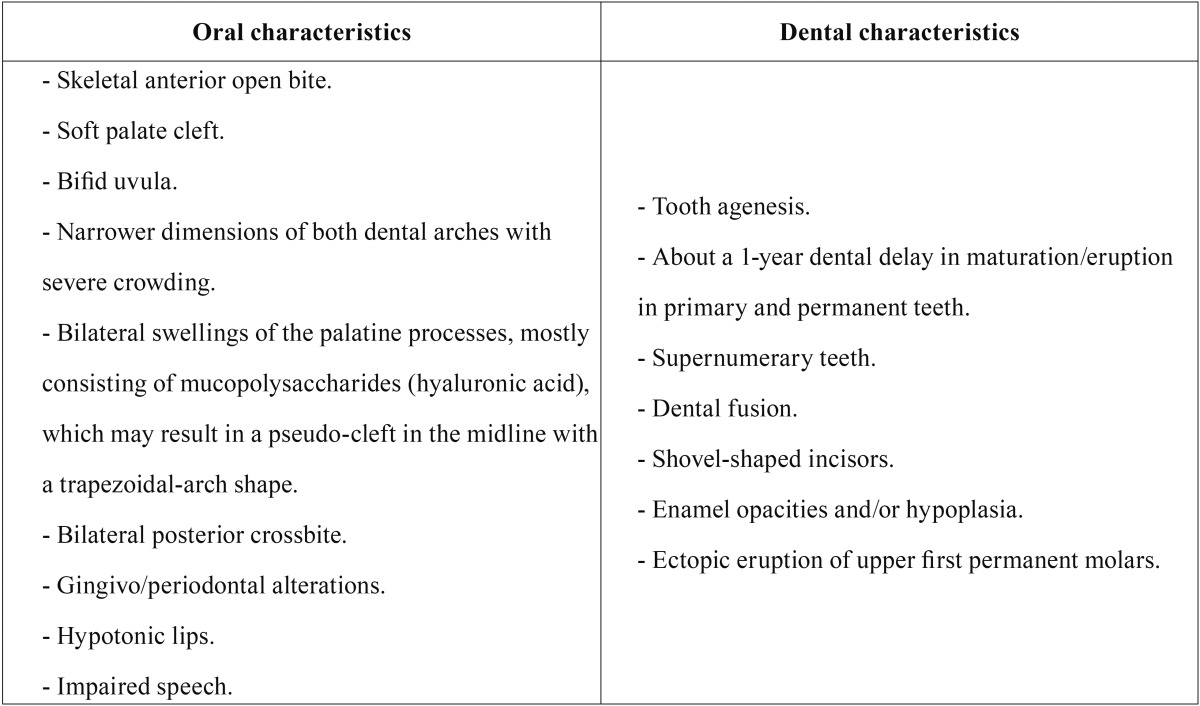
Apert Syndrome (AS) key oral/dental characteristics. Findings collected from some selected articles through the present systematic review.

Prevalence of the syndrome has been estimated to be between 1/65,000 and 1/200,000 newborns, without predilection by gender (11,17,18). AS has an autosomal dominant pattern of inheritance, associated with advanced paternal age, maternal infections, maternal drug consumption, and cranial inflammatory processes (18). More than 98% of cases are caused by *Fibroblast Growth FactoR (FGFR2)* gene-specific missense mutations at chromosome 10q25-10q26, which are exclusively paternal in nature (2,6,15,19,20). In this regard, it has been mentioned that the probability of a second child being affected is 1%, and that a person with AS has a 50% risk of having a child with the syndrome (8). The FGFR are a family of mitogenic signaling molecules that play an important role on the control of cell proliferation and survival (1); thus, in AS, fibroblasts are not able to produce the essential fibrous material in several craniofacial tissues, including bone sutures and cartilage, and during tooth formation and regeneration (4,18,20-22); thus, the mutated *FGFR2* gene may influence the dental abnormalities observed in AS (21).

AS can be confounded with another four similar craniosynostoses or skeletal dysplasias, such as Crouzon, Pfeiffer, Jackson-Weiss, Saethre-Chotzen, Beare-Stevenson, Carpenter, Vogt cephalodactyly, *cloverleaf* skull, and *FGFR3* coronal synostosis syndromes (7,14). Therefore, molecular genetic mapping of specific FGFR gene mutations or prenatal sonographic detection of structural abnormalities is recommended to confirm the diagnosis (4,23). Generalization of typical features associated with AS can be made to some extent, but each affected child exhibits a unique presentation, which requires being taken into account when preventive and therapeutic strategies are developed (11). Hence, the aim of the present review was to present the main results from a systematic review of the literature on AS, and to provide a brief description of female patient aged 4 years 6 months with AS.

## Material and Methods

For performing this systematic review, the authors strictly followed PRISMA (*Preferred Reporting Items for Systematic Reviews and Meta-analyses*) and Cochrane Collaboration criteria, designed especially for defining the following items: clinical question; literature search; inclusion/exclusion criteria; study selection; quality assessment, and information extraction and interpretation (24).

- Literature search strategy and results

An exhaustive Web literature search of relevant references on AS in Pediatric Dentistry was conducted in April/May 2016 by three authors, with the posing of the following posed clinical research question: What are the best oral/craniofacial diagnostic and management approaches for children and adolescents with Apert syndrome? Then, five electronic databases were exhaustively explored (publication dates from 1996–2016), restricted to the English and Spanish languages, in MEDLINE (via PubMed), EMBASE (Elsevier Science), Google Scholar, Cochrane Library (CENTRAL), and Scielo. Eligible methodological designs of published papers comprised narrative or systematic reviews, clinical trials, prospective or retrospective cohort and case-control studies, and clinical case reports. The patient population included subjects between 0 and 18 years of age. Thus, the following search algorithm (including keywords, entry MeSH-terms, Boolean operators, and filters) was developed:

((“acrocephalosyndactylia”[MeSH Terms] OR “acrocepha-losyndactylia”[All Fields] OR) (“apert”[All Fields] AND “syndrome”[All Fields]) (OR “apert syndrome”[All Fields]) OR (“acrocephalosyndactylia”[MeSH Terms] OR “acrocephalosyndactylia”[All Fields] OR) (“apert’s”[All Fields] AND “syndrome”[All Fields]) (OR “apert’s syndrome”[All Fields]) OR (“craniosynostoses”[MeSH Terms] OR “craniosynostoses”[All Fields] OR “craniosynostosis”[All Fields])) AND ((“paediatric dentistry”[All Fields] OR “pediatric dentistry”[MeSH Terms] OR) (“pediatric”[All Fields] AND “dentistry”[All Fields]) (OR “pediatric dentistry”[All Fields]) OR) (“dental care for children”[MeSH Terms] (OR (“dental”[All Fields] AND “care”[All Fields] AND “children”[All Fields]) (OR “dental care for children”[All Fields] OR) (“dentistry”[All Fields] AND “children”[All Fields]) (OR “dentistry for children”[All Fields])) AND ((“humans”[MeSH Terms] AND (“infant”[MeSH Terms] OR) “child”[MeSH Terms] OR “adolescent”[MeSH Terms])).

The list obtained of detected titles and abstracts was reviewed in detail to select the appropriate articles. These articles were then downloaded in full text. Additionally, a hand search was performed along the reference lists from each chosen full-text manuscript; to discard any repeats, careful further exploration was carried out. The final articles included were independently screened in a critical manner by two authors in order to evaluate their methodological quality and/or internal validity (risk of bias). Any discrepancy was resolved by discussion with the participation of a third author. Then, the information extracted from these papers was pertinent to the fundamental clinical matters of AS applicable to dentistry practice as follows: Disorder epidemiology; diagnostic process (including genetics and imaging); patient’s general features; craniofacial and oral characteristics, and general/dental management, with a pre-designed form employed for this purpose.

## Results

**Figure 1:**
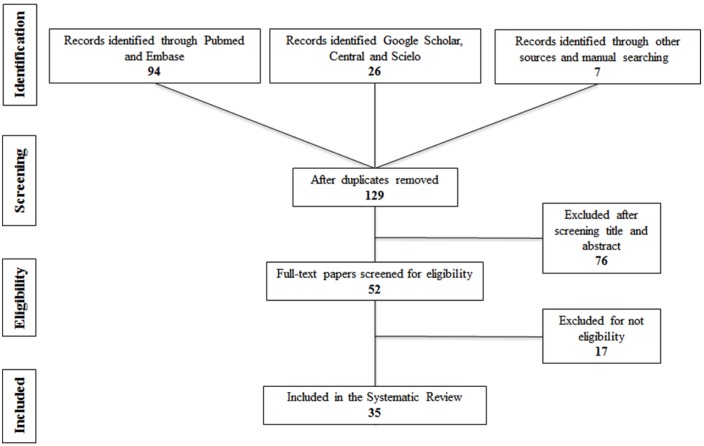
Flow-chart diagram of included articles.

The search of the electronic literature identified a total of 129 potential citation references. After reviewing the titles and abstracts of these articles, 77 clearly did not meet the desired criteria and were discarded. The full-texts of the remaining 52 citations were critically reviewed. Finally, 35 relevant papers were identified for inclusion in the present systematic review; these were then classified, according to publication/topic type, into four categories ( Table 2) (1,2-6,9,12,14,18-20,22,23). The majority of these were cross-sectional studies, and one article was in Spanish language (19) ( Table 3) (7,8,10,11,13,15-17,21,35,36). Figure 1 details the entire process of the article search and selection.

**Table 2:**
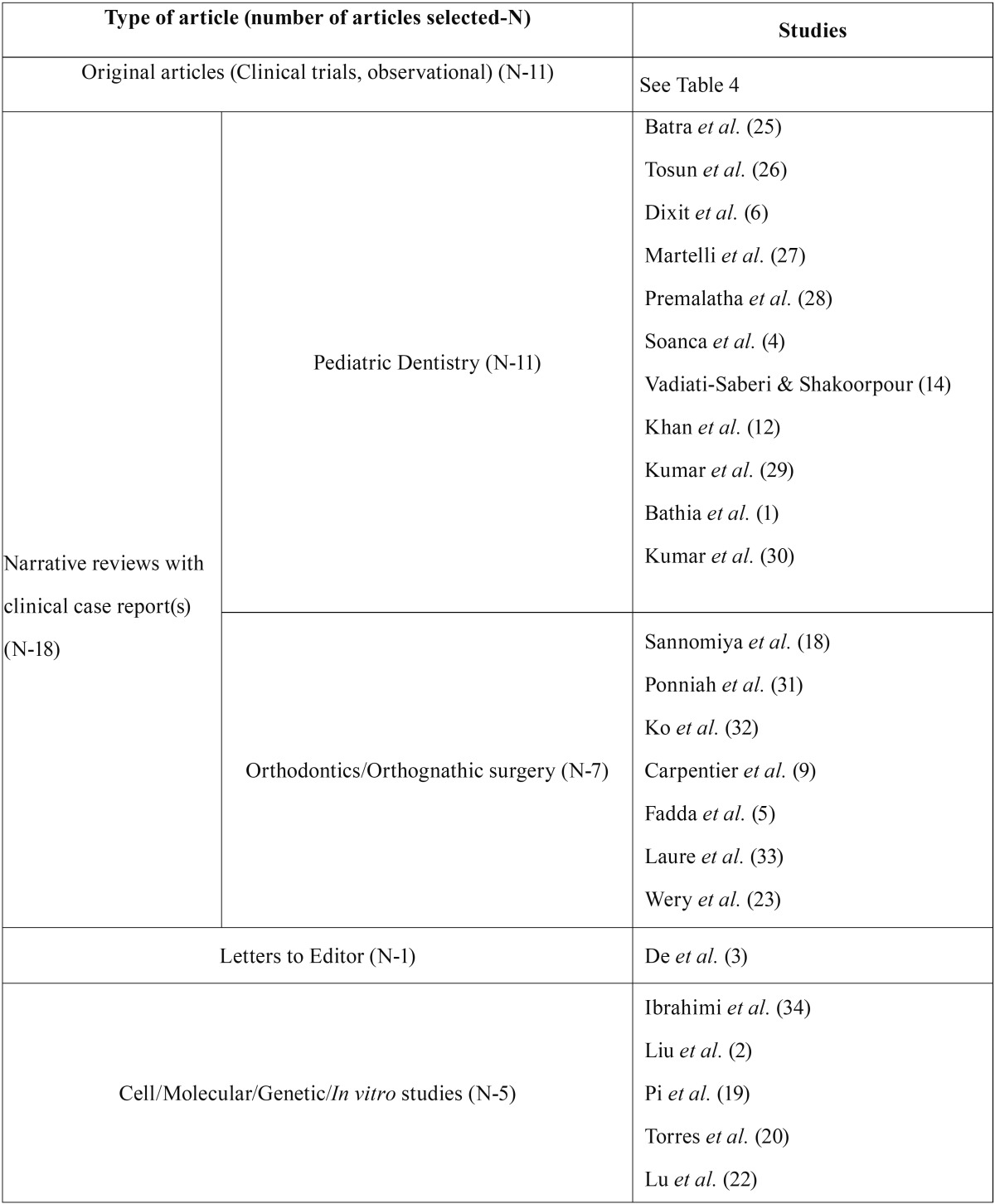
Classification of articles selected by the authors for the present systematic review.

**Table 3:**
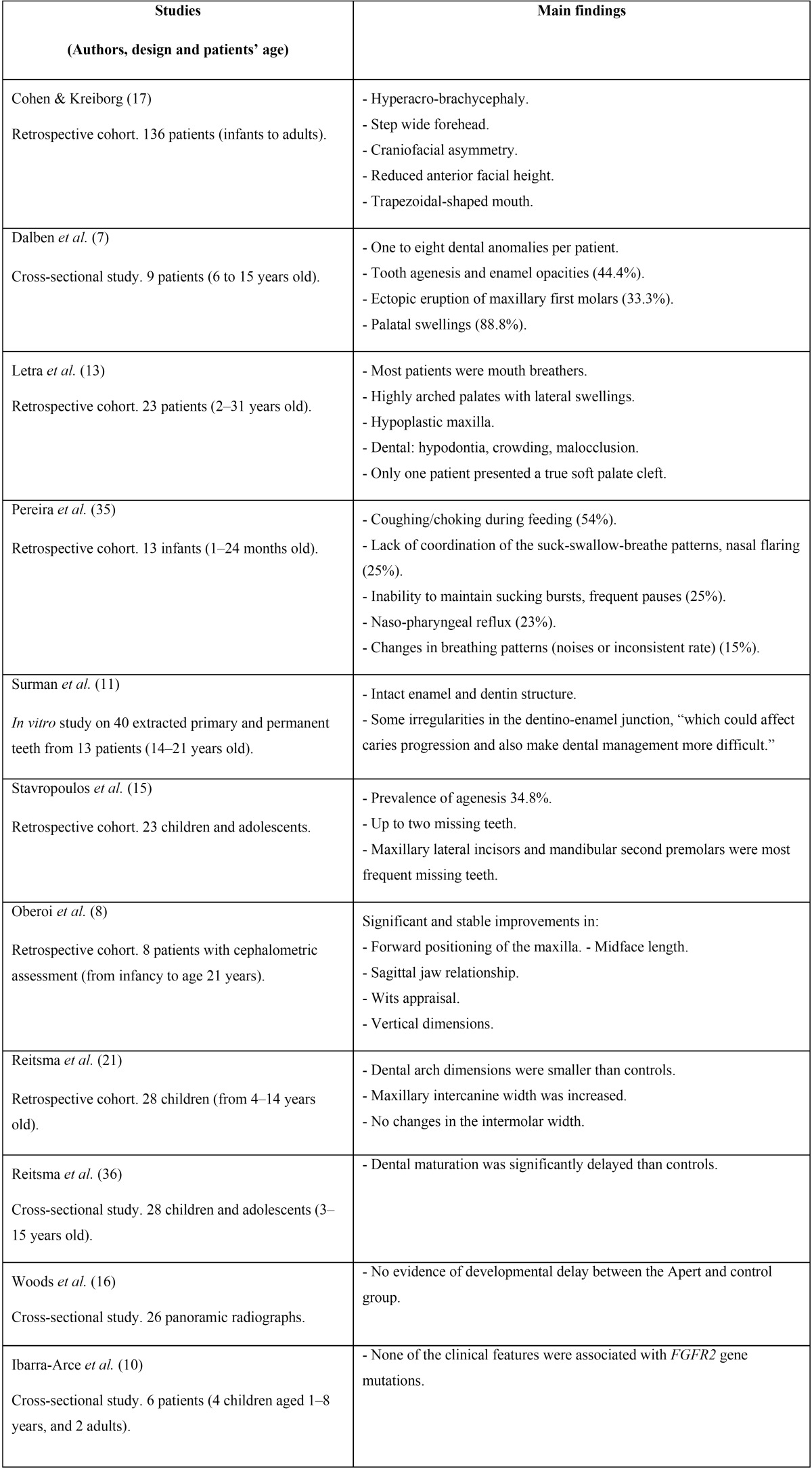
Main characteristics of the selected interventional/observational articles.

## Discussion

According to these systematic review findings, pediatric AS presents distinctive clinical and radiological features, together with usual orofacial anomalies, particularly midface undergrowth. Further, another interesting finding is the fusion of two or more cervical vertebrae, which occurs in 68% of affected patients (17). Although the majority of the articles included in the review provided consistent clinical information, we encountered, however, a few contradictory findings regarding the prevalence of oral anomalies; for example, while Reitsma *et al.* (36) mention significant dental maturation delay when compared with controls, Woods *et al.* (16) did not find any difference between patients with AS and the control group. This inconsistency may be due to different methodological characteristics employed in both studies.

The complex relationship between supporting basal maxillary and mandibular bones, as well as the pseudo or real cleft palate (with a reported prevalence of 25 and 75% in patients with AS), represent significant challenges for conventional dentistry practitioners (4). The majority of these concerns are related with esthetics, breathing, bottle-feeding, and swallowing difficulties in infants. In addition, affected children with limited arm and hand mobility are considered at high risk of caries, because this restriction makes it difficult to perform and maintain proper oral hygiene (8,26); dental practitioners can simplify this task by suggesting floss holders, electronic brushers, or home fluoride rinses, and by asking parents to help their children when necessary (26). Taken together, these problems may affect long-term systemic/oral health, growth, and development (6,35); furthermore, extensive structural and functional impairments can be life-threatening (8). Therefore, the dental management of patients with AS requires an integrated care team to care for the patient from infancy to adulthood, under the guidelines from worldwide accepted current protocols designed specifically for the oral/craniofacial area, which include preventive, restorative, endodontic, orthodontic/orthopedic, and orthognathic surgical approaches. Dentistry practitioners should comprise a crucial component of these care teams.

Prior to performing any dental procedure in a patient with AS, it is essential for the dentist to be capable of recognizing the disorder early, and then, the dentist must know the child’s craniofacial and oral therapeutic needs based on his/her unique combination of features (6). To carefully develop an individualized comprehensive treatment plan, a collaborative dental team should be integrated by different specialists. From birth, the oral and general management of children with AS is divided into supportive and reparative treatments ( Table 4) (25).

**Table 4:**
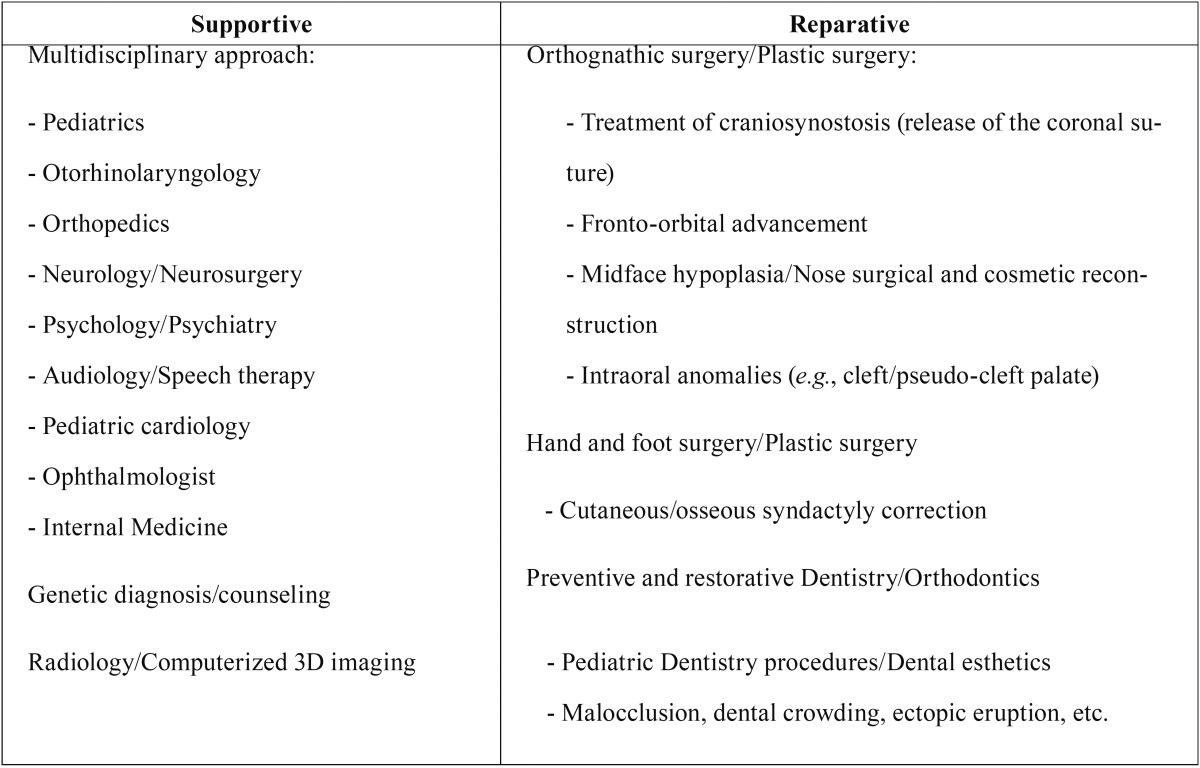
Types of treatment in children with Apert Syndrome (AS) Adapted from Batra *et al.* (25).

Thus, and according to Khan *et al.* (12), craniotomy to treat craniosynostosis and brain compression is usually performed at 6 months of age, corrective surgery for hand/foot digit syndactyly is carried out between the year 1 and 3–4 years of age, midface and palate corrections at 4–6 years, and orthognathic surgery/orthodontics, during the permanent dentition stage or after completion of growth. However, several interceptive or corrective fix/removable orthodontic appliances may already be placed during the primary and mixed-dentition stages (1).

Pediatric Dentistry practitioners can initiate their patient’s professional oral care very early, even before the child’s birth, by advising parents regarding AS treatment alternatives and nutritional or hygienic practices. Once the first teeth have erupted, it is very rewarding to plan frequent examinations, hygiene prophylaxis, fluoride treatments, pit/fissure sealants, and preventive or interceptive orthodontic approaches (26). Some of these procedures have been or will be applied in our patient with AS.

When recognized in the patient as early as 3 months of age, surgical treatment of the craniofacial region and fingers/toes may result in significant functional and cosmetic improvement (6). However, when the AS syndrome is diagnosed at later ages or treatment is delayed, many complications may arise, including the abnormal neurological development present in mental deficiency. With age, facial malformations, pseudo-prognathic mandible or class III malocclusion tends to increase and maxilla slants down posteriorly; in addition, finger function deteriorates with impeded skeletal growth and additional deformities. Fortunately, the majority of children with AS do not require any special dietary recommendations or activity/sport restrictions (1,6). On the other hand, the non-surgical manipulation of AS will be possible in the future, for example, by employing selective inhibitors of the FGFR-kinase domain (4,12,34).

**Figure 2:**
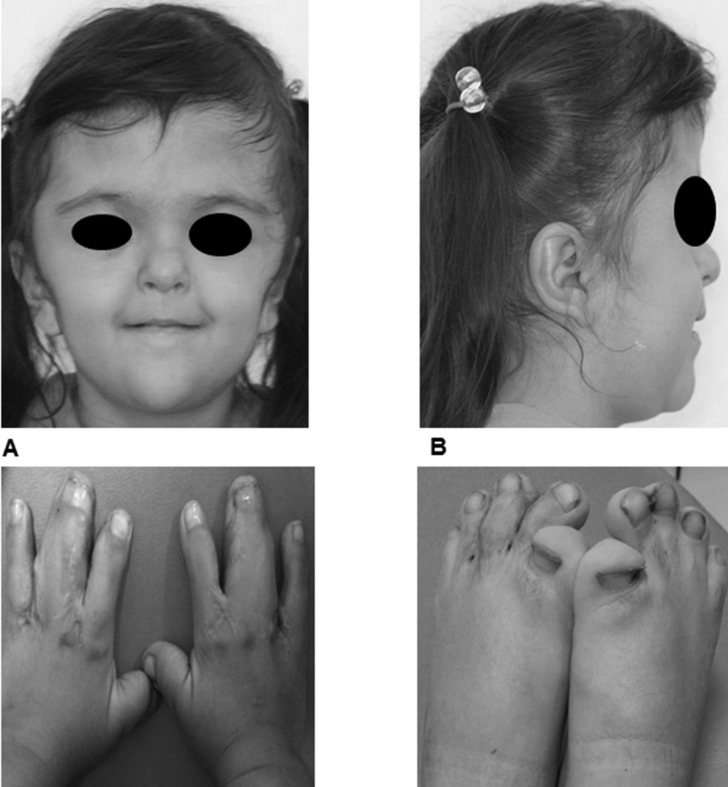
A. Face front. B. Face profile. C and D. Skin syndactyly in both hands and right foot. Scars are from previous reparative surgeries.

**Figure 3:**
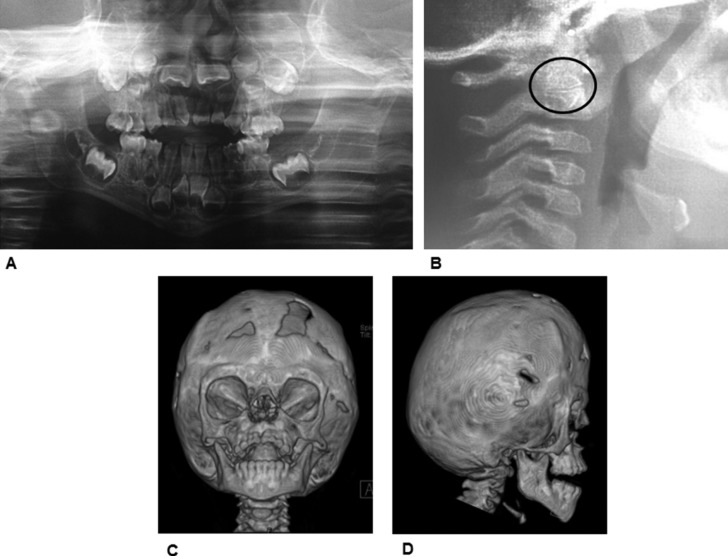
A. Panoramic view. Note the abnormal eruptive process of both permanent upper central incisors. B. Note the partial fusion between C2 and C3 body vertebrae, in their distal portions. Skull three-Dimensional (3D) Computed Tomography (CT) views. C. Front D. Right. Hypoplastic midface and skeletal anterior open bite are evident.

As a complement of this systematic review, we will briefly describe here a characteristic case of a 4.5-year-old girl with a diagnosis of AS, who was referred to the Pediatric Dentistry Postgraduate Program Clinic in the spring of 2016, with the chief complaint of a severe anterior open-bite. The child exhibited some typical AS craniofacial features as follows: abnormal head shape; webbing of central fingers in both hands and feet; shorter upper limbs, and mild developmental delay. She was the fourth child born to non-consanguineous parents and was diagnosed as suffering from AS during the month 1 of age. Prior to attending this dental visit, the patient had been undergone reconstructive cranioplasty for the coronal suture to reduce intracranial pressure, and both hands and left foot plasties to resolve partial cutaneous syndactyly. The girl exhibited poor manual dexterity for performing adequate oral hygiene. Other features were buccal breathing and slightly nasal speech. The patient displayed brachycephalic skull with short anteroposterior diameter, flat occiput, midface hypoplasia, frontal prominence, flat/steep forehead, hypertelorism, straight facial profile, and retruded lips (Figures 2A and 2B), as well as partial cutaneous webbed digits in hands (involving middle and annular fingers) (Figure 2C) and feet (with short and inward big toes) (Figure 2D). There was no other apparent congenital malformation and systemic examination revealed no hearing, neurological, cardiovascular, or internal-organ abnormalities. A palatal primary mesiodens root remnant was present. The palate was V-shaped and with a high arched vault, with pseudo-cleft in the posterior half, due to lateral swellings. A severe anterior open bite (around 9 mm) was exhibited, with thrusting and normal mouth opening. Additionally, there was complete primary dentition with interdental spaces in the lower arch due to absence of lateral incisors (primary and permanent), and moderate anterior crowding in upper arch, with exaggerated mesial steps. Mild dental-plaque deposits, several caries cavities, and white spots were present. Soft tissues were clinically normal. Extraoral radiographs (lateral skull and panoramic views) and a skull three-dimensional (3D) Computed Tomography (CT) scan were obtained (Figures 3A–3D). These revealed typical features, such as fused coronal sutures, brachycephalic skull, flat occiput, elongated/flat forehead, and partial fusion of vertebrae C2-C3, in their distal portions. The panoramic view exhibited no marked differences between dental age and eruption status and agenesia of both permanent lower lateral incisors.

## Conclusions

AS-type craniosynostosis comprises a major medical condition involving the craniofacial complex and the oral cavity, with significant related morbidity. Therefore, well-informed dentistry practitioners must play a crucial role in bucodental preventive/restorative management and other special needs of oral care. These tasks strongly contribute to the well-being of patients affected with Apert syndrome.
